# Achillin Increases Chemosensitivity to Paclitaxel, Overcoming Resistance and Enhancing Apoptosis in Human Hepatocellular Carcinoma Cell Line Resistant to Paclitaxel (Hep3B/PTX)

**DOI:** 10.3390/pharmaceutics11100512

**Published:** 2019-10-04

**Authors:** Jessica Nayelli Sanchez-Carranza, Leticia González-Maya, Rodrigo Said Razo-Hernández, Enrique Salas-Vidal, Ninfa Yaret Nolasco-Quintana, Aldo F. Clemente-Soto, Lucero García-Arizmendi, Mariana Sánchez-Ramos, Silvia Marquina, Laura Alvarez

**Affiliations:** 1 Facultad de Farmacia, Universidad Autónoma del Estado de Morelos, Av. Universidad 1001, Col. Chamilpa, Cuernavaca 62209, Morelos, Mexico; jessica.sanchez@uaem.mx (J.N.S.-C.); letymaya@uaem.mx (L.G.-M.); aldoclemente13@hotmail.com (A.F.C.-S.); lucero.gariz@gmail.com (L.G.-A.); 2 Centro de Investigaciones Químicas-IICBA, Universidad Autónoma del Estado de Morelos, Av. Universidad 1001, Col. Chamilpa, Cuernavaca 62209, Morelos, Mexico; nqny11@hotmail.com (N.Y.N.-Q.); marianasan_06@hotmail.com (M.S.-R.); 3 Centro de Investigación en Dinámica Celular-IICBA, Universidad Autónoma del Estado de Morelos, Av. Universidad 1001, Col. Chamilpa, Cuernavaca 62209, Morelos, Mexico; rodrigo.razo@uaem.mx; 4 Departamento de Genética del Desarrollo y Fisiología Molecular, Instituto de Biotecnología, Universidad Nacional Autónoma de México, Cuernavaca 62209, Morelos, Mexico; esalas@ibt.unam.mx; 5 Facultad de Ciencias Químico Biológicas, Universidad Autónoma de Sinaloa. Av. de las Américas y Blvd. Universitarios S/N, Culiacán 80010, Sinaloa, Mexico

**Keywords:** Achillin, Chemosensitization, multidrug resistance, paclitaxel, hepatocellular carcinoma, P-glycoprotein

## Abstract

Multidrug resistance (MDR) has become a major obstacle in the treatment of cancer, and is associated with mechanisms such as increased drug outflow, reduction of apoptosis, and/or altered drug metabolism. These problems can be mitigated by the coadministration of agents known as chemosensitizers, as they can reverse resistance to anticancer drugs and eventually resensitize cancer cells. We explore the chemosensitizing effect of Achillin, a guaianolide-type sesquiterpene lactone isolated from the Mexican medicinal plant *Artemisia ludovisiana*, to reverse MDR in Hep3B/PTX cells of hepatocellular carcinoma, which present resistance to paclitaxel (PTX). Achillin showed an important effect as chemosensitizer; indeed, the cytotoxic effect of PTX (25 nM) was enhanced, and the induction of G2/M phase cell cycle arrest and apoptosis were potentiated when combining with Achillin (100 μM). In addition, we observed that Achillin decreases P-gp levels and increases the intracellular retention of doxorubicin in Hep3B/PTX cells; in addition, homology structural modeling and molecular docking calculations predicted that Achillin interacts in two regions (M-site and R-site) of transporter drug efflux P-glycoprotein (P-gp). Our results suggest that the chemosensitizer effect demonstrated for Achillin could be associated with P-gp modulation. This work also provides useful information for the development of new therapeutic agents from guaianolide-type sesquiterpene lactones like Achillin.

## 1. Introduction

Hepatocellular carcinoma (HCC) is one of the most common tumor types worldwide, and with mortality rates similar to incidence [1], it is one of the most difficult cancers to treat. Cisplatin and doxorubicin are recommended treatments for HCC and have been widely studied; however, most cases are ineffective [2]. The use of microtubule-directed agent is an alternative with a high therapeutic potential in HCC. However, the multidrug resistance (MDR) phenomenon, which is acquired after exposure to a chemotherapeutic agent, including PTX, has become a major obstacle in the treatment of HCC [3,4,5,6,7]. Various mechanisms have been attributed to MDR, such as increased drug outflow, increased repair of DNA damage, reduced apoptosis, and/or altered drug metabolism [8]. P-gp, a trans-membrane glycoprotein, is an important member of the ATP-binding cassette transporter family, which function as pumps to extrude anticancer drugs from cancer cells, and is thereby closely related to the multiple resistance phenotype in cancer treatment. Moreover, several studies have reported the activation of P-gp in hepatocellular carcinoma cells resulting in reduced accumulation of chemotherapeutics and leads to resistance against some of the currently available anticancer drugs such as 5-fluorouracil (5-FU) [9] and Sorafenib [10] 

Among the strategies to reverse the MDR phenotype, the inhibition of P-gp function and expression are essential in the search for effective anticancer drugs [11]. The ability to modulate MDR has been seen complicated because many human tumors simultaneously exhibit multiple resistance mechanisms [12].

These problems could be mitigated by the coadministration of substances that inhibit MDR transporters as P-gp [13]. Such agents are known as chemosensitizers, as they can reverse resistance to anticancer drugs and eventually resensitize cancer cells to antineoplastic drugs, increasing the intracellular concentration of these drugs by inhibition of the P-gp efflux function [14,15]. 

However, although there are several compounds attributed with an effect on the function of Pg-p, the use of clinical trials using MDR inhibitors has had limited success [16]. Difficulties with inhibitors are mainly due to their toxicity, drug interactions, and clinical trial design problems [17]. 

Natural products are investigated by many researchers as MDR modulators for their low toxicity and potent selective behavior; reports show that coadministered MDR modulators compete with cytotoxic agents for binding to the active site of the membrane transporters and reduce drug efflux. The search for molecules based on natural products is important to reverse drug resistance during cancer therapy [18].

Secondary metabolites from plants, such as alkaloids, phenolics, terpenoids, and sesquiterpene lactones [19,20], can be substrates or inhibitors of P-gp and have been administered as chemosensitizers in combination with an antineoplastic agent and reversing resistance of cancer cells [21]. Sesquiterpene lactones constitute a group of C 15 terpenoids with a γ-lactone function, which represent the active components of many medicinal plants; they exhibit chemopreventive effects, which suppresses the growth of cancer cell lines [22,23]. Reports show that sesquiterpene lactone-like compounds have an important effect as chemosensitizers and that in combination with an antineoplastic agent the effect of this agent is potentiated in the induction of apoptosis and decrease metastasis [24]. For the abovementioned, in this study, we explore, for the first time, the chemosensitizing effect on human hepatocellular carcinoma cell line resistant to paclitaxel (Hep3B/PTX) of Achillin, a guaianolide-type sesquiterpene lactone present in some plants [23,24,25,26], isolated, in this work, from the Mexican medicinal plant *Artemisa ludoviciana* (Asteraceae).

Therefore, we show the ability of Achillin to reverse PTX resistance, by increasing the intracellular accumulation of doxorubicin and enhancing cellular death by apoptosis in the resistant to PTX hepatocarcinome cell line (Hep3B/PTX). Finally, a computational molecular docking study showed that Achillin interacts in two regions (M-site and R-site) of transporter drug efflux P-gp with higher affinity.

## 2. Materials and Methods 

### 2.1. Drugs and Chemicals

The Minimum Essential Medium was purchased of American Type Culture Collection (ATTC, Manassas, VA, USA). Verapamil, doxorubicin, and PTX were purchased from Sigma Aldrich (St. Louis, MO, USA), and glutamine and fetal bovine serum (FBS) were purchased from Thermo Fisher Scientific, Inc. (Waltham, MA, USA). Other reagents were of the highest analytical grade.

### 2.2. General

Achillin was isolated by means of open-column chromatography (CC). The isolation procedure and the purity was verified by thin-layer chromatography (TLC, 60F_254_ plate, Merck, Darmstadt, Germany), visualized by means of UV light, and sprayed with ammonium ceric sulfate (Ce(SO_4_)_2_·2(NH_4_)_2_SO_4_·2H_2_O. All ^1^H, ^13^C, DEPT, and 2D NMR (HSQC) were recorded on a Bruker AVANCE III HD 500 MHz equipment at 500 and 125 MHz, respectively, using CDCl_3_ with tetramethylsilane (TMS) as the internal standard.

### 2.3. Plant Material

*Artemisia ludovisiana* was collected in Cuernavaca Morelos, México. The plant was identified by the Biol. Gabriel Flores Franco and deposited at the HUMO Herbarium of the Universidad Autónoma del Estado de Morelos (UAEM), México, with the voucher number 33,913.

### 2.4. Isolation of Achillin

**Figure 1:**
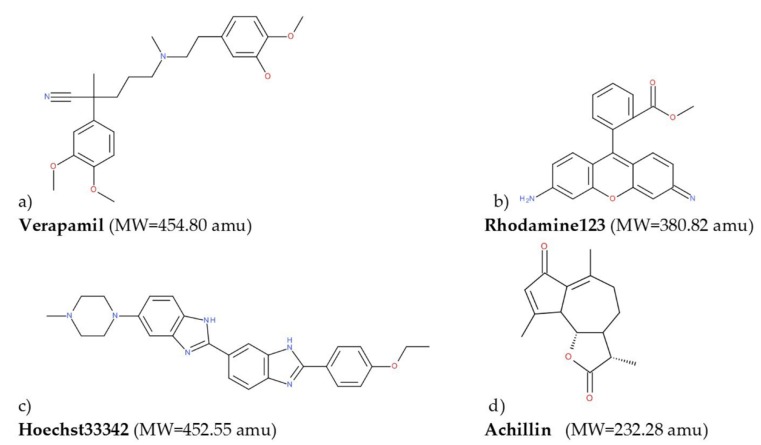
2D representation of the molecules used for molecular docking calculations: (**a**) Verapamil, (**b**) rhodamine123, (**c**) Hoeschst33342, and (**d**) achillin.

The dry and ground leaves of *Artemisia ludoviciana* (123.4 g) were extracted three times with methanol:water (95:05) by sonication; each extraction lasted 30 min. The extracts were combined and concentrated in a rotary evaporator, and a yellow residue was obtained (12.8 g). The methanol extract was fractionated into an open chromatographic column previously packaged with silica gel (230 g, 70–230 mesh; Merck) and eluted with a gradient of *n*-hexane/CH_2_Cl_2_/MeOH (20:80:00, 10:90:00, 00:100:00, 00:95:05, 00:90:10, 00:85:15, 00:75:25, and 00:00:100); 98 fractions of 100 mL were collected. The fractions were gathered according to their similarity observed in thin layer chromatography (TLC) in 5 groups: FrA-1-14 (1.32 g), FrB-16-26 (1.25 g), FrC-27-31 (1.06 g), FrD-40-87 (6.21 g), and FrE-87-103 (2.87 g). Fraction FrD-40-87 (4.2 g) was purified by column chromatography (Ф 4.5 mm × 70 cm) and packed with 130 g of silica gel; using an isocratic system prepared with *n*-hexane: CH_2_Cl_2_ (10:90), 38 fractions of 100 mL were obtained. Fractions 12-26 eluted with CH_2_Cl_2_:MeOH (95:05) showed a single component; finally, 1.25 g of the major component was obtained as fine needles. It was identified as Achillin [α]_D_: + 159 (c 0.8, MeOH) by direct comparison of its physical and spectroscopic properties of NMR with those of the literature [26,27] (Figure 1d). This compound was isolated from *Achillea santolina* and *Artemisia princeps* (Asteraceae) [26,28]. Copies of original spectra are available from the author of correspondence.

### 2.5. Chemosensitizing Potential to Paclitaxel

The hepatocellular carcinoma cell line Hep3B was obtained from ATCC (Manassas, VA, USA), and the Hep3B/PTX cell line was developed from the parental Hep3B cells by stepwise selection for resistance with increasing concentration of PTX. Briefly, cells were incubated with PTX just below their respective IC_50_ and the concentration was gradually increased from 10 to 350 nM. Cells were continuously cultured in the presence of PTX to maintain the acquired resistance. We showed that P-gp mRNA levels of Hep3B/PTX is higher than in Hep3B cells in this experiment Figure S1. We also included an immortalized human hepatocytes cell line (IHH) as a control of noncancerous cells [29]. All cells were grown in Minimum Essential Medium (ATCC, Manassas, VA, USA) and supplemented with BFS 10% and with 2 mM glutamine; all cultures were incubated at 37 °C in atmosphere of 5% CO_2_.

The chemosensitizing potential of Achillin on Hep3B and Hep3B/PTX cell lines was determined by assessing the cell viability using the MTS assay, for which 8000 cells/well were seeded in a 96-well cell culture plate and treated for 48 h with the desired concentration of Achillin and PTX, alone or in combination. Also, the effect of Verapamil, a known P-gp inhibitor [30], in combination with PTX and Achillin in combination with doxorubicin were evaluated. For determining the number of viable cells in proliferation we used CellTiter 96^®^ AQueous One Solution Cell Proliferation Assay kit (Promega, Madison, WI, USA), following the manufacturer’s instructions. Cell viability was determined by absorbance at 450 nm using an automated ELISA reader (Promega, Madison, WI, USA). Stock solutions of all compounds were prepared in DMSO at a maximum concentration of 0.5%. The experiments were conducted by triplicate in three independent experiments. Data were analyzed in Prism 5.0 statistical program (Graphpad Software Inc., La Jolla, CA, USA) and the IC_50_ were determined by regression analysis.

#### Analysis of Combination Effects

For analysis of the effect of combination, the following methods were applied. First, the combination index (CI) was calculated: it represents a general expression of drug interactions in pharmacology, and determining CI values is a simple way to quantify either synergism or antagonism. It is calculated as follows,

$$\mathrm{CI}=\frac{C\left(A,\;X\right)}{\mathrm{IC}\left(X,\;A\right)}+\frac{C\left(B,\;X\right)}{\mathrm{IC}\left(X,B\right)}$$

where *C* (*A*, *X*) and *C* (*B*, *X*) represent the concentrations of drug *A* and drug *B*, respectively, used in combination to produce mean effect *X* (IC_50_). IC (*X*, *A*) and IC (*X*, *B*) represent the median effect (IC_50_) values for single drugs *A* and *B*. The CI quantitatively describes synergism (CI < 1), additive effect (CI = 1), and antagonism (CI > 1) [31,32].

Furthermore, dose reduction indexes (DRI) were used to determine if a combination could lead to a reduction of the drug dose. DRI values are calculated as follows,

$$\mathrm{DRI}=\frac{{\mathrm{IC}}_{50}\left[\mathrm{drug}\;\mathrm{alone}\right]}{{\mathrm{IC}}_{50}\left[\mathrm{drug}\;\mathrm{in}\;\mathrm{combination}\;\mathrm{with}\;\mathrm{the}\;\mathrm{partner}\;\mathrm{drug}\right]}$$

### 2.6. Cellular Doxorubicin Retention Assay

To assess P-gp function inhibition by Achillin, we examined the cellular retention of doxorubicin a fluorescent P-gp-substrate, in Hep3B/PTX and Hep3B cells. In this study, 2.5 × 10^5^ cells were cultured for 24 h, and subsequently cells were either pre-incubated for 2 h 37 °C with control medium or medium containing Verapamil 20 μM (positive control) or Achillin (100 μM). Next, the cells were loaded with doxorubicin (20 μM) for 2 h at 37 °C in the presence or absence of these inhibitors; finishing the treatment time, the culture medium was removed, and cells were trypsinized followed by harvesting and washing twice with ice-cold PBS. The intracellular mean fluorescence intensity associated with the concentration of doxorubicin was analyzed by flow cytometry (Becton Dickinson, FACS Calibur, San Jose, CA, USA). Data analysis was performed using Cell Quest software (Tree Star, Inc., Ashland, OR, USA). The experiments were conducted by triplicate in three independent experiments.

### 2.7. Cell Cycle Analysis

The chemosensitizing effect of Achillin in combination with PTX on cell cycle progression was determined by flow cytometry. The Hep3B/PTX cells (3.0 × 10^5^ per well) were seeded into 6-well plates, and then treated with Achillin 100 µM, PTX 25 nM, and a combination of Achillin 100 µM + PTX 25 nM. Following treatments (24 h), the cells were trypsinized and collected into single cell suspensions, centrifuged, and fixed in cold ethanol (70%) overnight at −20 °C. The cells were then washed twice with PBS, resuspended in 500 μL PBS containing 60 μg/mL DNase-free RNase A (Sigma Aldrich, St. Louis, MO, USA) and 50 μg/mL propidium iodide (IP). The percentage of cells in G1, S, and G2 phases was analyzed with a flow cytometer (Becton Dickinson, FACS Calibur, San Jose, CA, USA); the number of cells analyzed for each sample was 10,000. The experiments were conducted by triplicate in three independent experiments. Data obtained from the flow cytometer were analyzed using the FlowJo Software (Tree Star, Inc., Ashland, OR, USA) to generate DNA content frequency histograms, and to quantify the number of cells in the individual cell cycle phases. The results were summarized in bar charts arranged by cell line, compound and cell cycle phase.

### 2.8. Flow Cytometric Apoptosis Assay

Apoptosis was evaluated using Annexin V Apoptosis Detection Kit FITC (Thermo Fisher Scientific, Waltham, MA, USA). The Hep3B/PTX cells (3.0 × 10^5^ per well) were seeded into 6-well plates and then treated separately with Achillin 100 µM, PTX 25 nM, and a combination of Achillin 100 µM + PTX 25 nM. After 48 h, the cells were harvested and washed twice with ice-cold PBS (0.01 M, pH 7.2). After 5 min of centrifugation at 200× *g*, Annexin V/FITC and PI double-staining were performed according to the manufacturer’s instructions. Cell apoptosis was analyzed on a FACS can flow cytometer (10,000 cells for each sample). Annexin V-positive, PI-negative cells were scored as apoptotic. Double-stained cells were considered either as necrotic or as late apoptotic. Data obtained from the flow cytometer were analyzed using the FlowJo Software.

### 2.9. Immunofluorescence

For P-gp expression and localization analysis, 10^4^ cells were added in 24-well culture plates containing glass slides and allowed to attach overnight at 37 °C in atmosphere of 5% CO_2._ Then, cells treated with 100 μM Achillin or nontreated were fixed with 4% paraformaldehyde in PEM buffer (PIPES 100 mM pH 6.9, EGTA 5 mM, MgCl2 2 mM) for 15 min followed by 4% paraformaldehyde in NaHCO_3_ for 45 min. The cells were permeabilized with 0.1% Triton X-100 (Sigma Aldrich) in PBS 1X for 10 min and then incubated with the primary antibody mouse anti-P-gp (1:100, sc-55510 Santa Cruz Biotechnology, Dallas, TX, USA) overnight at 4 °C. The secondary antibody (anti-mouse Alexa 647 1:1000, A-21235, Molecular Probes, Thermo Fisher Scientific, Inc., Waltham, MA, USA) was added and incubated for two hours at 37 °C. The cells were stained with Hoechst 33258 (1:4000, H1398, Molecular Probes) for one hour, mounted, and imaged by confocal microscopy. The experiments were conducted by triplicate in three independent experiments.

For DNA staining with DAPI, 3.5 × 10^4^ Hep3B/PTX cells were added in 24-well culture plates containing glass slides and allowed to attach overnight at 37 °C in 5% CO_2_. Then, cells were treated with Achillin 100 µM, PTX 25 nM and a combination of Achillin 100 µM + PTX 25 nM. The cells were fixed with paraformaldehyde (PFA, 4% in PEM buffer). After 15 min, PFA/NaHCO_3_ was added and incubated for 45 min at room temperature. The slides were rinsed with PBS and treated with 0.1% Triton X-100 (Sigma Aldrich). The cells were stained with DAPI (1:5000, D3571, Molecular Probes Probes) for one hour, mounted, and imaged by confocal microscopy. The experiments were conducted by triplicate in three independent experiments.

### 2.10. RT-PCR

For the analysis of P-gp mRNA levels in Hep3B and Hep3B/PTX cells, 2 × 10^5^ cells were seeded and treated with Achillin 100 µM, PTX 25 nM, and combination of Achillin 100 µM + PTX 25 nM. Then RNA was isolated. The total RNA extraction was performed employing a Quick-RNA MiniPrep Kit (Zymo Research, Irvine, CA, USA), following the manufacturer’s instructions. RNA was quantified using NanoDrop^®^ ND-1000 (Thermo Scientific, Waltham, MA, USA), and the RNA content of the samples was normalized. The RT-PCR was performed using a One-Step RT-PCR Kit with Thermo-Start Taq (Thermo Scientific, Waltham, MA, USA) following the manufacturer’s instructions. The primer sequences for Bcl-2 were 5-CCC TCC AGA TAG CTC ATT-3 and 5-CTAGACAGACAAGGAAAG-3. The Bax primer sequences were 5-ATGGACGGGTCCGGGGAG-3 and 5-TCAGAAAACATGTCAGCTGCC-3. The P-gp primers were 5-ACCATGGATCTTGAAGGGGACC-3 and 5-CCTCCAGATTCATGAAGAACCC-3. The GAPDH primers were 5-CAAGGTCATCCATGACAACTTTG-3 and 5-GTCCACCACCCTGTTGCTGTAG-3. All primers were synthesized by IDT-Integrated DNA Technologies (Redwood, CA, USA), and the reaction products of the samples were analyzed in 1.5% agarose gel.

### 2.11. Caspases Activity

Hep3B/PTX cells (8000 per well) were seeded on 96-well plates and treated with Achillin 100 µM, PTX 25 nM, and a combination of Achillin 100 µM and PTX 25 nM. After treatment, the caspase 3/7 activity was determined using the luminescent Caspase-Glo^®^ 3/7 Assay (Promega, Madison, WI, USA) following the manufacturer’s instructions. The results were represented as relative units of luminescence and represented in graphs, the statistical analysis was performed using the Prism 5.0 statistical program and the test performed was the Student’s t-test with significant *p*-values < 0.05.

### 2.12. Computational Details

Achillin interaction with P-gp transporters was also explored by in silico methods, leading to an improved understanding of the molecular mode of action of this compound.

#### 2.12.1. Homology Structural Modeling

Given that a full 3D structure of the P-glycoprotein of human is unavailable, we needed to generate a representative structure of this protein by means of the homology modeling approach. We used the I-TASSER server to resolve this task; I-TASSER has been ranked as the No. 1 server in the CASP7, CASP8, CASP9, and CASP10 competitions [33,34,35]. We employed the sequence of the *human* P-glycoprotein (GenBank:AAA59575.1) from the Proteins-NCBI web server. In this work, we used two approximations for the homology modeling, one considering the sequence homology, and the other considering the structural features of the TM helices. For the first case, we employed the crystal structure of *mus musculus* P-glycoprotein (mP-gp) as a template (PDB:3G61) for the construction of the homology model, because of the high sequence homology (83%) between this protein and human P-glycoprotein (hP-gp), an approximation used by Namseok Kim et al. [36]. On the other hand, Safiulla Basha et al. used the crystal structure of *Caenorhabditis elegans* P-glycoprotein (cP-gp) as a template (PDB:4F4C) for the construction of the homology model, which has a sequence homology of 46% compared to hP-gp, because of the more reliable orientation of the TM3, TM4, and TM5 helices [37]. To validate our models, a further analysis with PROCHECK server was done Figure S2.

#### 2.12.2. Molecular Docking Calculations

**Figure 2:**
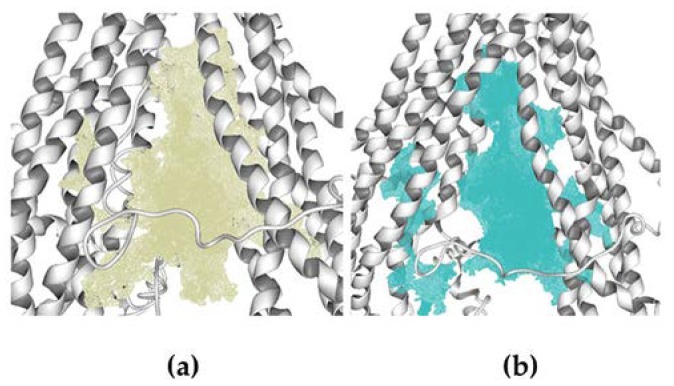
Cavities used to carry out the molecular docking calculations. (**a**) Cavity of homology model 1 (V = 3900). (**b**) Cavity of homology model 2.

All ligands used in this work Figure 1 were constructed with SPARTAN’18 [38]. An optimization geometry—without symmetry restriction employing the PM6 semiempirical approach—of all the molecules was carried out. To corroborate that the obtained structures were at a minimum in the potential energy surface, a vibrational frequencies calculation was done, see Figure 2.

Molecular docking calculations over human hP-gp structures generated in the homology modeling process were done in Molegro Virtual Docker (MVD) 6.0 [39,40]. The cavities were detected by the expanded van der Waals sphere method. The docking calculations were performed over the cavities formed by the twelve TM helices, this region is located between the cytosol and the inner membrane leaflet, with a volume of ~3900 Å^3^ and ~3700 Å^3^ for models 1 and 2, respectively, see Figure 2.

This region is where all the inhibitors of this protein bind, characterized by different drug-binding sites [41]. We employed the minimum energy geometries of all the molecules and evaluated different partial charges schemes (Molegro, electrostatic, and Mulliken). The residues within 3 Å of the cavity were set as flexible (138 residues); 2000 minimization steps for each flexible residue and 2000 steps of global minimization per run were set. The MolDock Simplex Evolution search function, based on an evolutionary algorithm, was used. A total of 50 runs with a maximum of 4500 iterations using a population of 50 individuals per run was set. To calculate the interaction energy, we used the scoring function Moldock Score [GRID]. The GRID resolution was set at 0.2 Å, and the search sphere is fixed at 28 Å. For the energy analysis of the ligand, the electrostatic internal interactions, internal hydrogen bonds and the sp2–sp2 torsions were considered. The method was validated by reproducing the experimental binding mode of the reference inhibitor within the 3G61 crystal structure to validate the mP-gp model, and 4F4C to validate the cP-gp model, with a Root Media Square Deviation (RMSD) of 2.1 Å, and 3.5 Å, respectively, see Figures S3–S5 in Supplementary Material).

### 2.13. Statistical Analysis

Statistical analysis was performed using GraphPad Prism 5 software (GraphPad Software, Inc., La Jolla, CA, USA). Data were expressed as mean ± SEM.

## 3. Results

### 3.1. Characterization of Resistant Hep3B/PTX Cells

Hep3B/PTX cells exhibited 14.08-fold resistance to PTX in comparison with its parental cell line Hep3B. The resistance index (RI), determined as the ratio of IC_50_ (concentration of PTX required to produce 50% inhibition) of the resistant Hep3B/PTX cells to the IC_50_ of the parental Hep3B cells is shown in Table 1.

**Figure 3:**
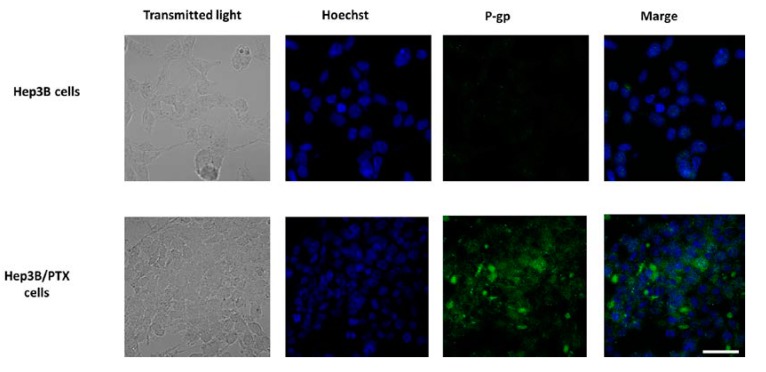
P-gp expression and localization by immunofluorescence microscopy in parental Hep3B and resistant Hep3B/PTX cells. The phase-contrast, P-gp (green), and Hoechst 33258 nuclear counter stain (blue). High levels of P-gp protein were localized in the plasma membrane of resistant cell Hep3B/PTX. Images were acquired at 40X objective plus a 2X zoom in confocal microscope settings. Scale bars, 40 μm.

Previous studies have confirmed that MDR in cancer cell lines and human tumor tissues is most often associated with the overexpression of the P-gp, resulting in decreased intracellular drug accumulation [9,10]. For this reason, we focus on the P-gp study and analyze whether the significant increase IC_50_ in the resistant cells could be associated with P-gp overexpression. The results showed high levels of P-gp were observed in Hep3B/PTX resistant cells compared to parental Hep3B cells Figure 3. These results suggest the implication of P-gp in the MDR phenotype of Hep3B/PTX cells.

### 3.2. Achillin Increases the Sensitivity of Hep3B/PTX Cells

**Figure 4:**
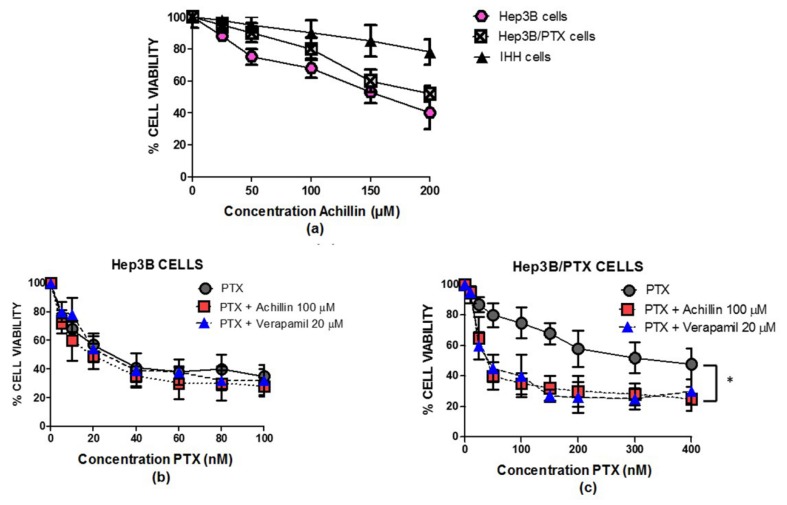
Antiproliferative activity of achillin and PTX alone and in combination in cell lines. (**a**) Antiproliferative effect at different concentrations of achillin on Hep3B, Hep3B/PTX, and IHH cells. (**b**) Antiproliferative effect at different concentrations of PTX, alone and in combination with 100 µM Achillin, or 20 µM Verapamil on Hep3B cells. (**c**) Antiproliferative effect at different concentrations of PTX, alone and in combination with 100 µM Achillin, or 20 µM Verapamil on Hep3B/PTX cells. The absorption values are represented in relation to drug-untreated culture cells (arbitrary value of 100). The results are the mean ± SD of at least three determinations. * *p* ≤ 0.05.

When examining comparative Achillin antiproliferative activity in concentrations between 25 and 200 μM, in parental (Hep3B), resistant (Hep3B/PTX), and immortalized human hepatocytes (IHH) cell lines (Figure 4a), Achillin did not show a significative change in reduction in the percentage of viable cells between Hep3B and Hep3B/PTX cells; nevertheless, this effect was lower in intensity in the noncancerous cells, IHH, showing selectivity for cancer cells.

Based on the above determined cytotoxic values in parental Hep3B cells and resistant Hep3B/PTX cells, the concentration of 100 μM (IC_20_) of Achillin was selected for the combination treatment assays with PTX in the following determinations of antiproliferative activity, cell cycle, and apoptosis induction in Hep3B/PTX cells. Then, we comparatively analyzed the effect of PTX at concentrations ranging from 10 to 400 nM; treatments with PTX alone caused a few pronounced drug-dependent decreases in the number of viable cells. Thus, treatment with 25 nM PTX caused approximately 15% decrease, whereas 400 nM PTX caused a 50% decrease in Hep3B/PTX cells. However, we could observe that combined treatment PTX (25 nM) plus achillin (100 μM) cooperated in more than additive manner to inhibit viability, suggesting a chemosensitivity effect of achillin. This result is comparable to the effect observed by the treatment of (PTX 25 nM) plus verapamil (20 μM), a chemosensitizing agent used as positive control Figure 4b.

The modulation of PTX cytotoxicity in drug-sensitive Hep3B and drug-resistant Hep3B/PTX cells by Achillin are shown in Table 2. The cytotoxicity of PTX was enhanced by achillin, the combination of these two compounds diminishes the IC_50_ from 352 ± 12 nM of PTX alone, to 38 ± 5 nM. The results showed a great similarity in the resistance reversal index (RRI) between the PTX/Achillin treatment (RRI = 9.1), compared to that observed by the treatment of the positive control PTX/Verapamil (RRI = 10). This is the first time that the drug resistance modulation activity of Achillin has been reported.

Based on the IC_50_ values determined above, two nontoxic concentrations (IC_20_, IC_40_) of Achillin were combined with PTX. The IC_50_ values of PTX alone and in combinations Achillin in Hep3B/PTX cell lines are recorded in Table 3. Furthermore, the combination indexes (CI) and the dose reduction indexes (DRI) for two-drug combinations were calculated.

As shown in Table 3, Achillin significantly decreases the IC_50_ value of PTX, thus showing chemosensitizing activity. The nature of the combinations was evaluated by CI analysis, as described before. In general, all the calculated CI values are below 1, meaning that the interactions are synergistic. Dose reduction is important, because it could lead to reduced toxicity and to maintained or increased main therapeutic efficacy. Favorable DRI values would be >1, whereas unfavorable ones would be <1.

On the ground of the here obtained results, the concentrations of 100 μM Achillin and 25 nM PTX were adopted for further experiments with Hep3B/PTX.

### 3.3. Cell Cycle Distribution

**Figure 5:**
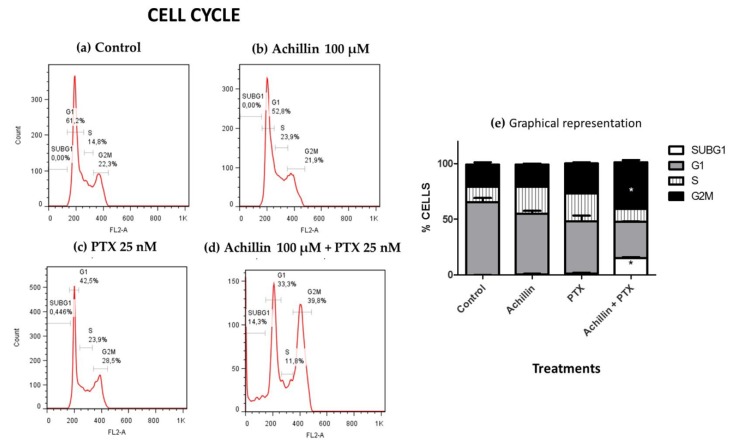
Cell cycle disruption by PTX antitumor drugs and Achillin. Figure 5(**a**–**d**) shows the percentages of Hep3B/PTX cells in the G0/G1, S and G2/M cycle phases and of cells with sub-G1 DNA content after different treatments. (**a**) Cells without treatment (control). (**b**) Achillin 100 μM. (**c**) PTX 25 nM. (**d**) Achillin 100 μM + PTX 25 nM. (**e**) Graphical representation of histograms and cell frequencies representative of three determinations. Achillin was applied 2 h before PTX. * *p* ≤ 0.05.

PTX is a potent microtubule-targeting agent known to cause mitotic cell cycle arrest (G2M phase) [42]. After establishing that achillin resensitizes cancer cells to PTX and potentiates its antiproliferative activity in resistant cells, our objective was to evaluate the effect of PTX in combination with achillin on the cell cycle progression in Hep3B/PTX cells. The results showed that Achillin (Figure 5b) and PTX (Figure 5c) had no significant effect on the Sub G1 and G2M phase of the cell cycle comparing them with the untreated cells. However, the achillin (100 µM) plus PTX (25 nM) treatment (Figure 5d), increased the proportion of cells in the G2/M phase from 28.5% to 39.8%, at the tested concentrations, this increase was statistically significant with respect to the treatment with only PTX, also an increase from 0% to 14% in the subG1 phase was observed, which is indicative of cell death (Figure 5e), this result suggests that Achillin potentiates the effect of PTX in Hep3B/PTX cells.

### 3.4. Effects of Achillin Plus PTX on Cell Death in Hep3B/PTX Cells

**Figure 6:**
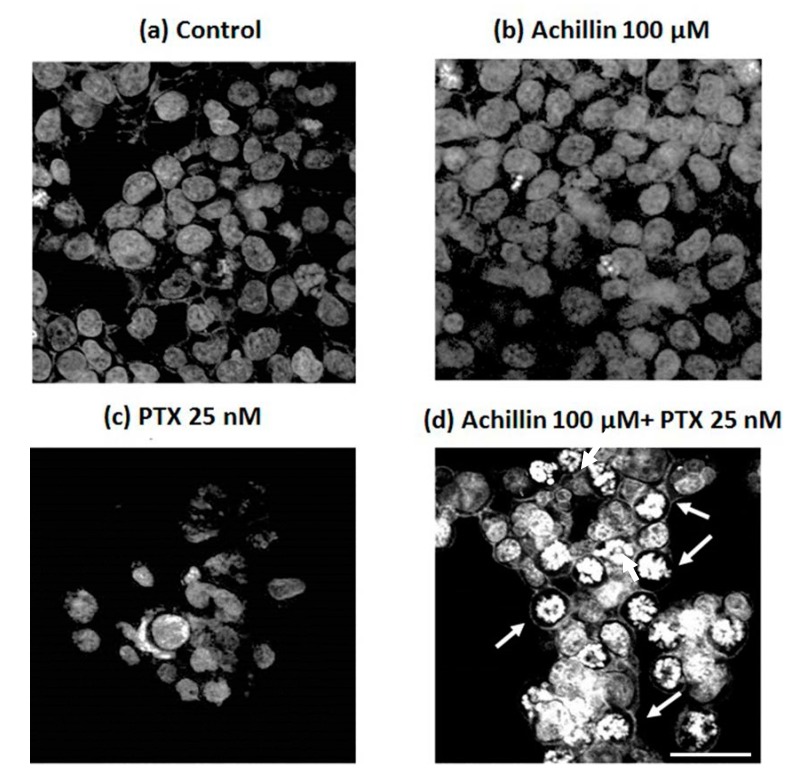
Confocal microscopy of DAPI-stained treated Hep3B/PTX cells. (**a**) Negative control, without treatment cells. (**b**) Achillin 100 μM. (**c**) PTX 25 nM. (**d**) PTX 25 nM + Achillin 100 μM. Images are representative of the three independent experiments. Images were acquired at 40X objective plus a 2X zoom in confocal microscope settings. Scale bars, 40 μm.

Due the obtaneid results on the potentiated effect of PTX by pretreatment with Achillin in Hep3B/PTX cells, on inhibition of cell viability, and increase in G2M phase of cell cycle. Next, we evaluated the combined effect of Achillin more PTX in the induction of apoptosis associated with changes in DNA fragmentation, and chromatin marginalization, which are characteristics of apoptosis. Accordingly, the DAPI fluorescence analysis (Figure 6) revealed apoptotic nuclear morphological changes, such as chromatin condensation and marginalization (arrows), were evident in cells exposed to Achillin 100 µM + PTX 25 nM treatment (Figure 6d), whereas in the treatment with PTX alone, minimal nuclear alterations in the cells (Figure 6c) were observed. These results suggested apoptosis in cells treated with Achillin before exposure to PTX.

### 3.5. Effect on PTX-Induced Apoptosis

**Figure 7:**
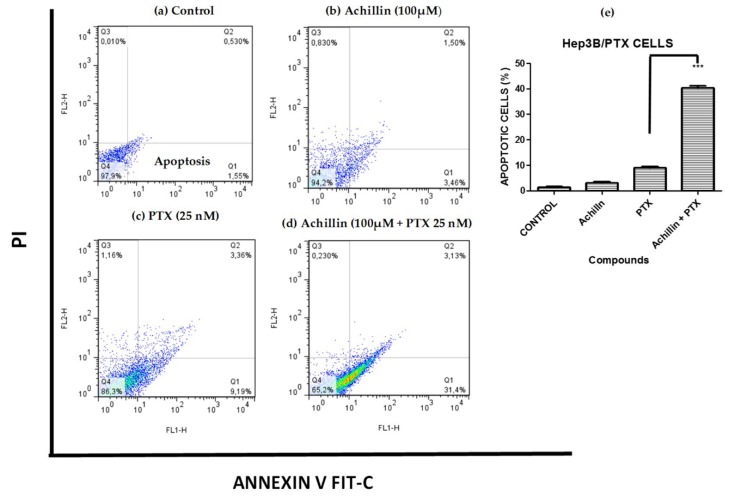
Cell death analysis in Hep3B/PTX cells by flow cytometry using Annexin V-FITC/PI double staining. (**a**) Untreated cells. (**b**) Achillin 100 µM. (**c**) PTX 25 nM. (**d**) Achillin 100 µM +PTX 25 nM. (**e**) Percentage of apoptotic cells, the data are expressed as the means ± S.E.M. of three independent experiments. Early apoptotic cells (annexin V-positive and PI-negative; lower right quadrants), late apoptotic cells (annexin V-positive and PI-positive; upper right quadrants), and necrotic cells (annexin V-negative and PI-positive; upper left quadrants). Statistical significance was determined by one-way ANOVA followed by Dunnett’s test, then compared to the control. *** *p* ≤ 0.001.

The regulation of achillin and PTX on induced apoptosis of Hep3B/PTX cells was evaluated by quantification of apoptotic cells. Phosphatidylserine externalization, a hallmark of early phase apoptosis, was analyzed using annexin V-FITC/PI double staining and flow cytometry. As shown in Figure 7, the percentage of apoptotic cells was 3.46% in treatment with 100 µM Achillin (Figure 7b) and 9.26% when exposed to 25 nM of PTX (Figure 7c). However, in the treatment of PTX 25 nM plus Achillin 100 µM, the percentage of apoptotic Hep3B/PTX cells was 34% (Figure 7d). The analysis of these results showed a statistically significant increase of 3.39-fold of the apoptotic population compared with 25 nM PTX treatment alone Figure 7e. Altogether, this suggests that Achillin enhances the apoptosis induction of PTX.

**Figure 8:**
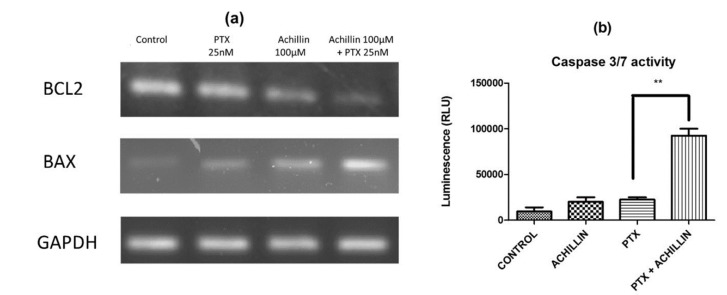
Apoptosis induction by PTX and Achillin either alone or in combination in Hep3B/PTX cells. (**a**) Effect of PTX 25 nM, Achillin 100 µM, and PTX 25 nM + Achillin 100 μM on mRNA expression levels of Bcl-2 and Bax in Hep3B/PTX cells. GADHP was used as an internal control. (**b**) Graphic representation of effect on caspases activity of PTX 25 nM, Achillin 100 µM, and PTX 25 nM + Achillin 100 μM in Hep3B/PTX cells. Achillin was applied 2 h before PTX.** *p* ≤ 0.05.

In this way, to determine whether the pure compounds induce apoptotic cell death by regulating antiapoptotic (Bcl-2) and proapoptotic (Bax) proteins in the hepatocellular carcinoma Hep3B/PTX cells, expression of Bax and Bcl-2 was analyzed by RT-PCR. Ours results showed a clear inhibition of Bcl-2 and an increase of Bax transcripts after the treatment with Achillin plus PTX (Figure 8a). Therefore, to support the previously observed effect on Bax/Bcl-2 expression, and knowing that the Caspases play a key role in the initiation and execution of apoptosis, the caspase 3/7 activity was evaluated on Hep3B/PTX cells after treatment with Achillin plus PTX Figure 8b. The results show a higher caspase 3/7 activity when compared to the non-treated control, as well as the treatments with PTX, and Achillin alone.

### 3.6. Effect of Achillin on P-gp Protein Levels in Hep3B/PTX Cells by Immunofluorescence

**Figure 9:**
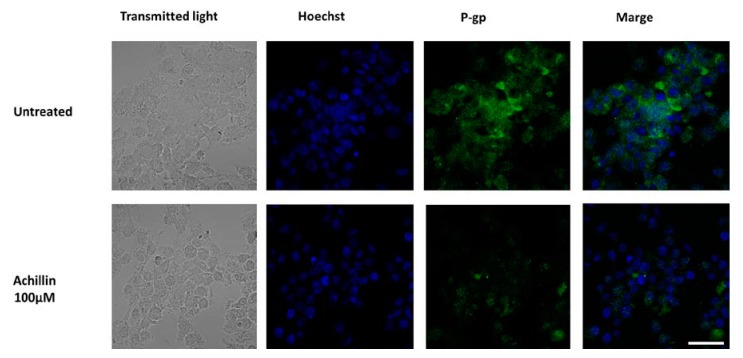
Effect of Achillin (100 µM) on P-gp expression and localization by immunofluorescence microscopy in Hep3B/PTX resistant cells. The phase-contrast, P-gp (green), and Hoechst 33258 nuclear counter stain (blue). Images were acquired at 40X objective plus a 2X zoom in confocal microscope settings. Scale bars, 40 μm.

P-gp plays an important role in determining the concentration of substrates inside the cells; P-gp located in the plasma membrane directly effluxes cytotoxic drugs from cells and, therefore, reduces their concentration [37]. It had been previously shown by others studies that chemotherapeutics or P-glycoprotein inhibitors affected P-gp expression levels in cancer cells and that this can be a cause of re-sensitization of MDR cells [43,44]. We evaluated the effects of Achillin on P-gp levels using immunofluorescence analysis and found that cancer resistant cells exposed to Achillin result in decrease in P-gp levels Figure 9. This suggests that the chemosensitizing effects presented by Achillin could be associated with the modulation of P-gp expression. Moreover, we proceeded to evaluate its effect on the efflux function of the P-gp transporter.

### 3.7. Effect on Cellular Doxorubicin Retention by Achillin

**Figure 10:**
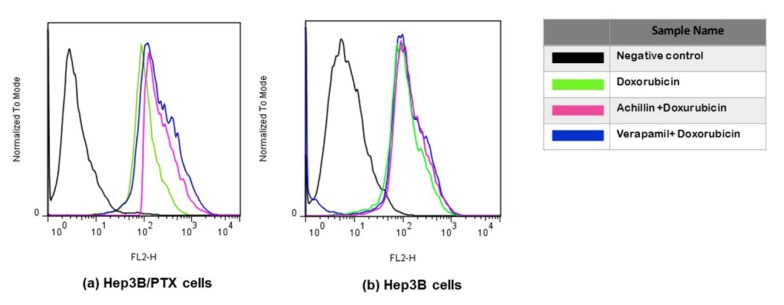
Doxorubicin accumulation. Flow cytometry overlay of Doxorubicin fluorescence intensity after treatments: (**a**) Hep3B/PTX, (**b**) Hep3B cells, and Hep3B/PTX cells. The cells treated with DMSO were used as the negative control (black line); Doxorubicin 20 μM (green line), pretreatment 100 μM Achillin plus Doxorubicin 20 μM (pink line), and pretreatment 20 μM Verapamil plus Doxorubicin 20 μM (blue line) in Hep3B cells.

Doxorubicin is also a good substrate for MDR-associated P-glycoprotein, and agents that inhibit P-glycoprotein function have been found to increase accumulation of doxorubicin in drug-resistance cells. Therefore, the ability of Achillin to inhibit P-glycoprotein function was evaluated by determining the intracellular doxorubicin-associated fluorescence intensity (FI) in Hep3B/PTX cells. Flow cytometry was used to detect fluorescence of doxorubicin (as explained in the methods section). As shown in Figure 10a, the intracellular doxorubicin-associated FI was increased in the presence of the reference P-gp inhibitor Verapamil (20 μM), which is known to fully inhibit P-gp activity. Achillin 100 μM also shifted the fluorescence intensity of doxorubicin rightwards. These results suggest that Achillin may inhibit the function of P-gp, which could explain the potentiated cytotoxic effect and apoptosis induction of paclitaxel in combination with Achillin in Hep3B/PTX resistant cells.

On the other hand, we observed that the level of accumulation of doxorubicin hardly varied in the nonresistant Hep3B cells, regardless of whether it was treated with or without prior treatment of Achillin and Verapamil Figure 10b, which supported the idea that the PTX-induced cytotoxicity increased by Achillin towards Hep3B/PTX could be attributed to modulation of activity of the P-glycoprotein.

**Figure 11:**
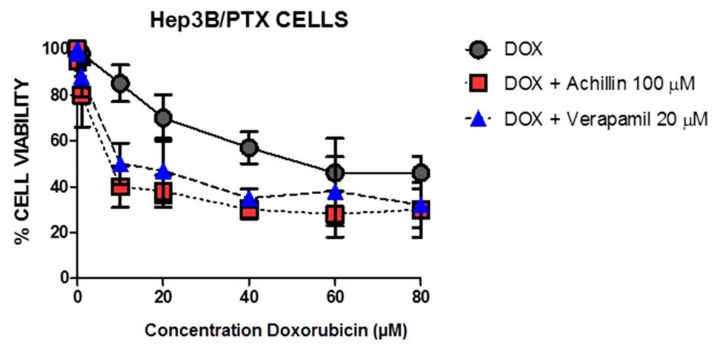
Effect at different concentrations of doxorubicin alone and in combination with 100 µM Achillin or 20 µM Verapamil on Hep3B/PTX cell proliferation. The absorption values are represented in relation to untreated cultures (arbitrary value of 100). The results are the mean ± SD of at least three determinations.

To clarify the inhibitory effect of Achillin on the drug efflux activity of P-gp, we perform the cytotoxic assay using doxorubicin and Achillin in Hep3B/PTX cells. It is known that compounds with chemosensitivity capacity are associated with the modulation of the outflow activity of the transporters, reversing MDR, and consequently increase the intracellular concentration of chemotherapies and enhance the cytotoxicity of antineoplastics [45]. Figure 11 shows significant inhibition in cell proliferation when the cells were treated with doxorubicin in combination with achillin or verapamil, suggesting inhibitory effect of Achillin on the drug efflux activity of P-gp.

### 3.8. Theoretical and Computational Results

Molecules that are P-gp substrates and modulators can act as inhibitors (impairing P-gp mediated uptake or efflux) or simply substrates (translocating across membranes via P-gp), but one drug can also have overlapping roles [46]. To predict possible interactions and binding modes between Achillin and P-gp protein, an exhaustive molecular docking analysis was done.

#### 3.8.1. Homology Structural Modeling

We obtained quite acceptable models from our two approximations—mP-gp and cP-gp—with C-score values of 1.14 and 1.61, respectively; the homology model confidence increases according to the I-TASSER C-score from −5 to 2, that is, higher values make more reliable models. Additionally, we used the PROCHECK server to validate our homology models based on their Ramachandran plots (Supplementary Materials Figures S3 and S4). The mP-gp model showed 84.3% of residues (970 amino acids) in the most favored region; 12% of residues (138 amino acids) in additional allowed region; 2.3% of residues (26 amino acids) in generously allowed region; and 1.5% of residues (17 amino acids) in the disallowed region. The cP-gp model showed 85.3% of residues (982 amino acids) in the most favored region; 11.1% of residues (128 amino acids) in additional allowed region; 2.4% of residues (28 amino acids) in generously allowed region; and 1.1% of residues (13 amino acids) in the disallowed region. According to these results, the best homology model for the hP-gp was obtained with the cP-gp approximation. Nevertheless, since the differences are not remarkable, and in order to obtain more diversity of the ligands biding results, based on two structural conformations of the hP-gp main chain, we decided to use the two homology models for the docking calculation.

**Figure 12:**
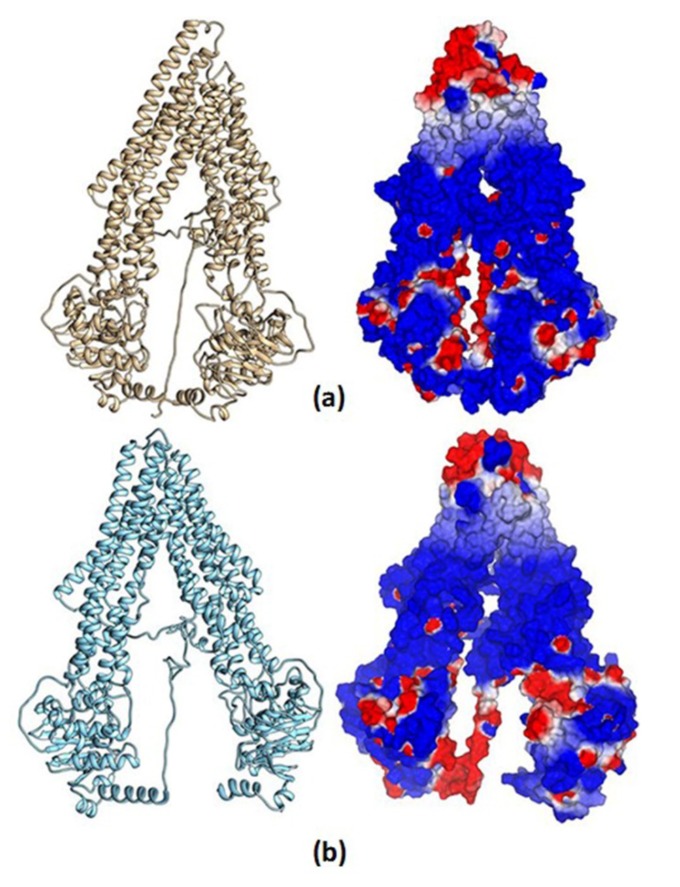
Human P-glycoprotein homology models and their molecular electrostatic potential map. (**a**) Homology model using mP-gp as template. (**b**) Homology model using cP-gp as template. Blue, red, and white colors indicate positive, negative, and zero MEP values, respectively.

In Figure 12, the total structure of the two hP-gp models and their Molecular Electrostatic Potential (MEP) are displayed. It can be observed that the structural differences of these models remain in the “hairpin” shape type formed by the twelve TM helices, where the mP-gp model possesses a more closed structure. To evaluate how these structural modifications can affect the interactions with each ligand, we calculated the MEP for each model. It can be appreciated that the electronic features of the protein are mostly conserved despite the structural differences, that is, the MEP regions are similar for the two models: blue-, red-, and white-colored regions, which represent positive, negative, and zero MEP values, respectively. Thus, the docking results can be related mostly to the cavity shape.

#### 3.8.2. Molecular Docking Calculations

**Figure 13:**
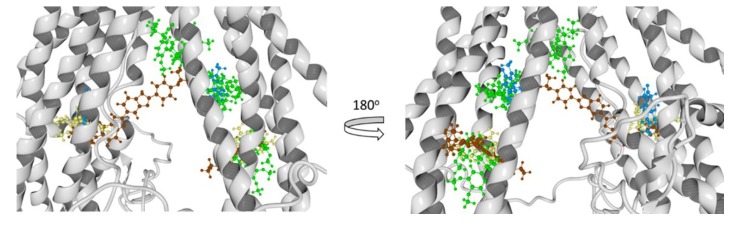
Molecular docking results of all the ligands in this work over the mP-gp model. Verapamil (green color), rhodamine123 (yellow color), Hoechst 33342 (brown color), and Achillin (blue color).

**Figure 14:**
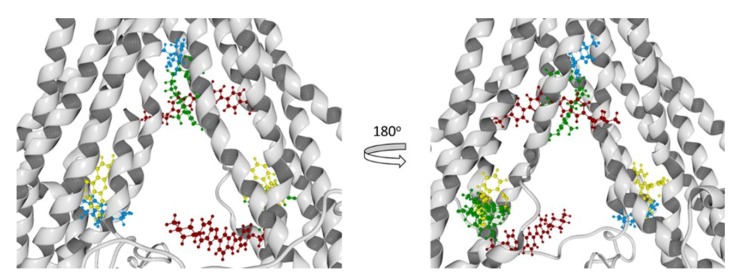
Molecular docking results of all the ligands in this work over the cP-gp model. Verapamil (green color), Rhodamine123 (yellow color), Hoechst 33342 (dark red color), and Achillin (blue color).

To have greater confidence in the results obtained by our molecular docking method, we evaluate it with three known P-glycoprotein-binding compounds: verapamil, Rhodamine123, and Hoechst 33342. As a crystal structure of one of the most-studied ligand binders of P-glycoprotein is unavailable, we based our results in the interaction residues of each ligand with the two models of hP-gp; many works have been done in this manner [36,37,41]. In Figure 13, the best poses of each ligand in this study over the mP-gp model are displayed. Also, in Figure 14, the best poses of each ligand in this study over the cP-gp model are shown.

**Figure 15:**
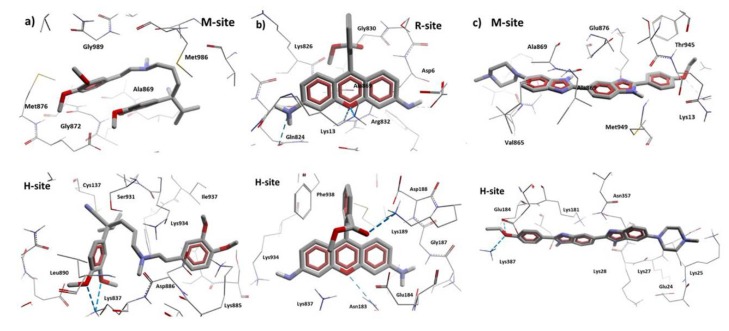
HB interactions of Verapamil, Rhodamine123, and Hoechst 33342 with cP-gp homology model. (**a**) Interactions of the two Verapamil poses with cP-gp model. (**b**) Interactions of the two Rhodamine123 poses with cP-gp model. (**c**) Interactions of the two Hoechst 33342 poses with cP-gp model. The HBs are displayed as blue dashes.

From the literature, we know that we can divide the great binding zone of the P-glycoprotein in three regions: M-site, R-site, and H-site. The M site is located next to the inner leaflet interface, the top of the cavity where all the TM helices flow. R-site is composed by TM helices 6, 9, and 12, and H-site is formed by TM helices 2, 3, 4, 10, and 11 [38]. From previous works, the location of Verapamil, Rhodamine123, and Hoechst 33342 and the most probable binding sites in the hP-gp are reported. According to this Verapamil preferably binds in the M-site, while Rhodamine123 binds in the R-site and Hoechst 33342 binds in the H-site [39,40,41]. From Figure 13 and Figure 14, it can be noted that Verapamil binds in the M-site and H-site in both hP-gp models (green-colored molecules). In the same way, Hoechst 33342 interacts in these two sites (M-site and H-site) in both hP-gp models. On the other hand, Rhodamine123 binds the R-site and H-site, as shown in Figure 13 and Figure 14. From these results, we observe that despite the structural difference in the main chain of the two hP-gp models, the best poses of each reference ligand bind in the same sites, with different pose conformation and interaction residues (they are mostly conserved). Because of this, we only analyzed the hydrogen bond (HB) interactions of the compounds with the cP-gp model (in Figure S6 the HB interactions with mP-gp are displayed). In Figure 15, the HB interactions of the three reference ligands with cP-model are shown.

According to our results, all the reference ligands bind in their respective binding sites and in an additional one: M-site for Hoechst 33342 and H-site for Verapamil and Rhodamine123. This is in accordance with previous works; as there is no crystal structure available, the ubication of the binding site of these molecules is still uncertain. In Table 4, the interaction residues of the cP-gp model with all the ligands in this study are displayed.

**Figure 16:**
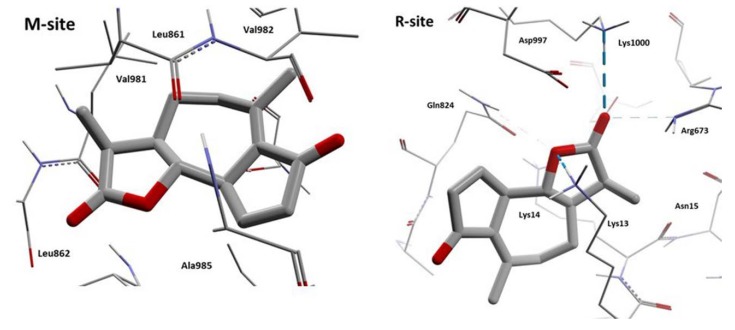
HB interactions of the two poses of Achillin with cP-gp homology model. The HBs are displayed as blue dashes.

According to these results, we can observe that Achillin interacts in two regions of the hP-gp protein (M-site and R-site). If we analyze the interaction residues and compare with the reference ligands, the number of similar interaction residues is with Rhodamine123 when Achillin interacts in the R-site. In Figure 16, the HB interactions of Achillin with the cP-gp model are shown.

From Figure 16 we can observe that Achillin forms HB interactions with cP-gp model, when it binds in the R-site, by means of its oxygen atoms. The great number of positive charged residues like Lys and Arg benefits the location of this molecule by increasing its binding stability. In addition, it can be noted that Achillin and Rhodamine123 have molecular similarity, both are small planar cyclic molecules with a small number of oxygen atoms.

In Table 5 the interaction energy (E_inter_) and ligand efficiency (LE) values of each ligand (both poses) with the cP-gp model are displayed; the ligand efficiency represents the coefficient of the E_inter_ and the total number of heavy atoms (all atoms in the molecule excluding hydrogen).

From Table 5 it can be noted that Achillin possess a good E_inter_ value compared to the rest of P-gp inhibitors. In addition, Achillin has a better LE value that all the reference inhibitors. LE is used to compare the interaction profile of different MW ligands and analyze the atom contribution of each ligand to the E_inter_.

## 4. Discussion

Reports show that sesquiterpene lactone-like compounds have an important effect as chemosensitizers and, when used in combination with an antineoplastic agent, the effect of this agent is potentiated in the induction of apoptosis.

In the present report, we investigated chemosensitizing potential of Achillin a natural sesquiterpene lactone isolated from the Mexican medicinal plant *Artemisia ludovisiana*, as a reversal agent to overcoming PTX resistance of Hep3B/PTX cells.

For such purpose, Hep3B/PTX cells were subjected to Achillin and PTX treatments alone and in combination. Cell proliferation assays showed that PTX effect on the inhibition of cell proliferation was clearly increased when the Hep3B/PTX cells were previously treated with Achillin. On the other hand, and to strengthen our results, we analyzed the effect of PTX on the cell cycle progression alone and in combination with Achillin; it is known that PTX is a microtubule-stabilizing agent, preventing the depolymerization of microtubules during mitosis, blocking cell cycle progression in the G2/M phase, and finally inducing apoptosis. In this study, Hep3B cells resistant to PTX at a concentration of 25 nM do not showed detention in G2/M phase of the cell cycle. However, when treated previously with Achillin, an increase in the G2/M phase was observed, confirming that Achillin chemosensitizes the cells to the effect of PTX.

Apoptosis assay demonstrated that Achillin enhanced apoptosis induction of PTX, an increase in apoptotic cells detected by Annexin V and nuclear fragmentation of chromatin detected by confocal microscopy was observed. Finally, RT-PCR analysis showed promotion of the expression of Bax and suppression of the expression of Bcl-2 and, further, an increase in caspases 3/7 activity. Taken together, these results demonstrate that Achillin increases chemosensitivity to PTX overcoming resistance and enhancing apoptosis in Hep3B/PTX cells.

Other reports show that sesquiterpene lactone-like compounds have an important effect as chemosensitizers, and that in combination with an antineoplastic agent the effect of this agent is potentiated in the induction of apoptosis. One example is the natural sesquiterpene lactone parthenolide who enhances sensitivity of human A549 cells by lowering the dose of oxaliplatin and developing induction of apoptosis [47]. While, in ovarian cancer resistant cells it was observed that combinations of the EPD lactone with paclitaxel increases the arrest of the cell cycle in the G2/M phase with respect to the effect observed only by the paclitaxel treatment [48]. Meanwhile other study showed the chemosensitizing capacity of this type of compounds that when combined with docetaxel decreased the metastasis in an in vivo model of breast cancer xenograft [48], and reverse vincristine resistance in colon carcinoma cells [49].

It is known that compounds with chemosensitivity capacity are associated with the modulation of the outflow activity of the transporters, potential substrates of P-gp reverse multidrug resistance, consequently, increase the intracellular concentration of chemotherapies and enhance the cytotoxicity of antineoplastic [45]. For example, in KB-C2 cells, carnosic acid sensitized vinblastine cytotoxicity by reversing multidrug resistance, suggesting inhibitory effects on anticancer drug efflux transporter P-gp and enhancement of the efficacy of vinblastine [50].

However, clinical trials using MDR inhibitors have had limited success [16]; with the exception of cyclosporine that was used to inhibit P-gp in patients with low-risk acute myeloid leukemia, resulting in significant gains in overall survival and no relapse [51]. Difficulties in clinical trials with inhibitors are mainly due to inhibitor toxicities, drug interactions and clinical trial design problems [9,10,11,12,13,14,15,16,17]. Verapamil is the prototype P-gp blocker [52], and it is known to cause serious and devastating immunosuppressive and cardiovascular effects [53,54].

P-gp has been a target for drug discovery for almost 40 years and despite these complications in the use of inhibitors, it does not diminish the impact or the importance that the search for effective P-gp modulators would have, which can be used in chemotherapies against cancer with favorable results in patients.

In the present study we showed the chemosensitizing effect of Achillin in resistant cells Hep3B/PTX. It is known that there are various transport proteins of the ABC superfamily, which include (P-gp/MDR1/ABCB1), multidrug resistance-associated protein-1 (MRP1), and the breast cancer resistance protein (BCRP), and the inhibition of these proteins is associated with MDR reversal. We focus on P-gp because it is the protein most frequently associated in several tumor types with the MDR phenomenon. Our results show that Achillin had a clear effect on the inhibition of P-gp transporter; however, the effect of Achillin on the modulation of other transporters was not evaluated, although reports show that compounds that modulate P-gp could have a similar effect with other ABC superfamily transporters; this should be corroborated [55,56].

Also, Achillin decreases P-gp expression levels and increases the intracellular accumulation of doxorubicin, and it is known that when P-gp function is modulated, the cellular retention of chemotherapeutics is increased; these compounds are considered substrates of P-gp [57,58], suggesting Achillin as a molecule candidate in the effective modulation of P-gp efflux function. Therefore, it was important to clarify the explicit binding sites of Achillin at P-gp, this protein contains more than one substrate binding domains, therefore, it is of relevant importance from the pharmacokinetic, pharmacological and toxicological perspectives.

According to our docking results, Achillin interacts in two regions (M-site and R-site) of transporter drug efflux P-gp with higher affinity, also showed a better LE value that all the reference inhibitors and substrate. It is known that substrates occupy different regions in the common drug-binding pocket. The binding of substrates with P-gp induces conformational changes in the protein, with a concomitant change of the ATPase activity of P-gp [59,60,61].

Another important point of our results is that Achillin lacks of hydrogen bond donors (HBD), has a small MW and a PSA value of 34.96 Å^2^, which make this molecule a good candidate for use as a molecular scaffold for the design of future P-gp inhibitors.

The use of natural molecules such as Achillin could be more advantageous versus conventional inhibitors of P-gp such as Verapamil, which has some limitations and always produce toxic effects towards normal cells. In this regard, due to its low toxicity, potency, and efficacy, and according to our docking results, selective behavior for binding to the active site of the membrane transporters and reduce drug efflux may exist.

## 5. Conclusions

Achillin, a natural sesquiterpene lactone, showed chemosensitizing capacity, potentiating cytotoxic activity and apoptosis induction of PTX. In addition, we observed that Achillin decreases the P-gp level and increases the intracellular accumulation of doxorubicin in Hep3B/PTX. Our computational molecular docking results predicted an affinity and binding interactions between Achillin and the transporter P-gp in M-site and R-site, which suggests that the here demonstrated chemosensitizer effect of Achillin could be associated with the modulation of P-gp a transporter drug efflux. This work also provides useful information for the development of new therapeutic agents from guaianolide-type sesquiterpene lactones like Achillin.

## Figures and Tables

**Table 1:** IC_50_ values of paclitaxel (PTX) in sensitive Hep3B and PTX-resistant Hep3B/PTX cell lines.

PTX IC_50_ nM	
PTX/Hep3B	Hep3B
352 ± 12 ^a^	25 ± 5 ^a^

^a^ Required amount of drug alone to reduce the growth of the cell lines by 50%.

**Table 2:** Modulation of PTX cytotoxicity in drug-sensitive Hep3B and drug-resistant Hep3B/PTX cell lines by Achillin.

Treatment	PTX ^a^IC_50_ nM (RRI) ^b^ Hep3B Cells	PTX ^a^IC_50_ nM (RRI) ^b^ PTX/Hep3B Cells
PTX alone	25 ± 5	352 ± 12 ^a^
PTX + Achillin 100 µM	19.5 ± 3 (1.28)	38 ± 5 (9.1)
PTX + Verapamil 20 µM	21.4 ± 4.2 (1.16)	33 ± 10 (10)

^a^ Required amount of drug alone to reduce the growth of the cell lines by 50%. ^b^ Resistance Reversion Index (RRI) = (IC_50_ of drug alone)/(IC_50_ of drug in combination with modulator).

**Table 3:** Cytotoxicity of PTX against Hep3B/PTX cells, either alone or combinations with Achillin. The IC_50_ (µM) are presented as the mean ± SD. CI values <1 signify synergism; =1: additive effects; >1: antagonism. 0.3 < CI < 0.7 means synergism (+++), 0.7–0.85 moderate synergism (++). NR = not relevant. DRI = dose reduction indexes.

Treatment	IC_50_ PTX	DRI	CI	Interpretation
PTX alone	0.352 ± 0.012	1	NR	NR
PTX + Achillin	-	-	-	-
100 µM (IC_20_)	0.038 ± 0.0091	9.2	0.5	+++
160 µM (IC_40_)	0.03 ± 0.007	7.3	0.88	++

**Table 4:** Interaction residues of the cP-gp model with all the ligands.

Molecule	M-site	R-site	H-site
Hoechst 33342	Thr945, Ala871, Met949, Glu875, Phe942, His61, Ala869, Gly989, Val991, Gln195, Met948, Val873, Gly872, Val865, Ser992, Ala947, Ile870, Val988, Leu843, Gln132, Met876, Ile868, Gln946	-	Glu29, Lys181, Asp177, Lys25, Asp26, Lys887, Lys28, Asn357, Glu184, Glu24, Lys27, Leu890, Asn183
Verapamil	Met986, Glu875, Met949, Gly989, Ile868, Met876, Ala985, Val982, Gly872, Val988, Ala869, Ala871, Val873, Ser952, Gln990, Leu3, Val865	-	Asp886, Lys934, Phe938, Ser931, Lys887, Glu184, Ala883, Cys137, Asn183, Leu879, Lys885, Ala935, Ser880, Ile937, Leu890, Asn930, Gln882, Gly141, Asp188, Ser180
Rhodamine123	-	Lys13, Asp997, Gln824, Ser831, Asp6, Ala823, Phe994, Gly9, Gly827, Leu236, Ile829, Ala828, Leu833, Arg673, Lys14, Gly830, Lys826	Glu184, Asp188, Lys189, Phe938, Lys877, Lys887, Cys137, Lys934, Asn183, Ile186, Asp886, Ser931, Ala140, Gly187, Val133, Val185, Ala883
Achillin	Leu861, Val982, Phe978, Leu862, Ile868, Ala985, Ser952, Ile847, Thr858, Phe983, Met986, Val865, Val981	Gln824, Lys13, Lys14, Ala823, Lys826, Asp679, Asp997, Arg673, Lys1000, Asn15, Phe16, Phe17, Ala828, Gly10, Gly827	-

**Table 5:** Interaction energy and ligand efficiency values (kcal/mol) of the cP-gp model with all the ligands.

Molecule	M-site	R-site	H-site
Hoechst 33342	E_inter_ = −81.53 kcal/mol LE = −2.40 kcal/atom	-	E_inter_ = −83.17 kcal/mol LE = −2.45 kcal/atom
Verapamil	E_inter_ = −99.18 kcal/mol LE = −3.00 kcal/atom	-	E_inter_ = −110.04 kcal/mol LE = −3.33 kcal/atom
Rhodamine123	-	E_inter_ = −71.97 kcal/mol LE = −2.77 kcal/atom	E_inter_ = −41.22 kcal/mol LE = −1.59 kcal/atom
Achillin	E_inter_ = −53.04 kcal/mol LE = −3.12 kcal/atom	E_inter_ = −84.93 kcal/mol LE = −4.99 kcal/atom	-

## Supplementary Materials

The following are available online at https://www.mdpi.com/1999-4923/11/10/512/s1. Figure S1: Analysis of mRNA levels of P-gp in Hep3B/PTX and Hep3B cells, Figure S2: Structural validation of the docking calculations, using the crystal structure of mus musculus P-glycoprotein (PDB: 3G61), Figure S3: Structural validation of the docking calculations, using the crystal structure of Caenorhabditis elegans P-glycoprotein (PDB: 4F4C), Figure S4: Ramachandran plot of mP-gp homology model, Figure S5: Ramachandran plot of cP-gp homology model, Figure S6: HB interactions of Verapamil, Rhodamine123 and Hoechst 33342 with mP-gp homology model.
