# Supplementary material for: Achillin Increases Chemosensitivity to Paclitaxel, Overcoming Resistance and Enhancing Apoptosis in Human Hepatocellular Carcinoma Cell Line Resistant to Paclitaxel (Hep3B/PTX)
Source: Pharmaceutics. 2019 Oct 4;11(10):512. doi: 10.3390/pharmaceutics11100512 (PMC6835644; doi:10.3390/pharmaceutics11100512)
Supplement: Supplementary file 1 [file pharmaceutics-11-00512-s001.pdf]

pharmaceutics

MDPI

Supplementary Materials: Achillin Increases Chemosensitivity to Paclitaxel, Overcoming Resistance and Enhancing Apoptosis in Human Hepatocellular Carcinoma Cell Line Resistant to Paclitaxel (Hep3B/PTX)

Jessica Nayelli Sanchez-Carranza, Leticia González-Maya, Rodrigo Said Razo-Hernández, Enrique Salas-Vidal, Ninfa Yaret Nolasco-Quintana, Aldo F. Clemente-Soto, Lucero García-Arizmendi, Mariana Sánchez-Ramos, Silvia Marquina and Laura Alvarez

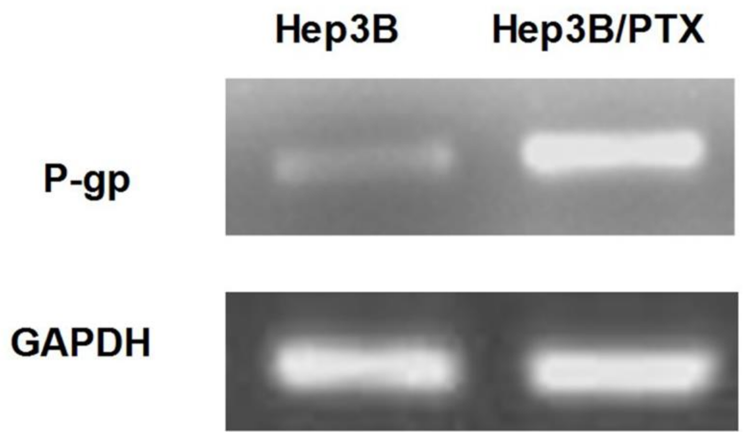

Figure S1. Analysis of mRNA levels of P-gp in Hep3B/PTX and Hep3B cells.

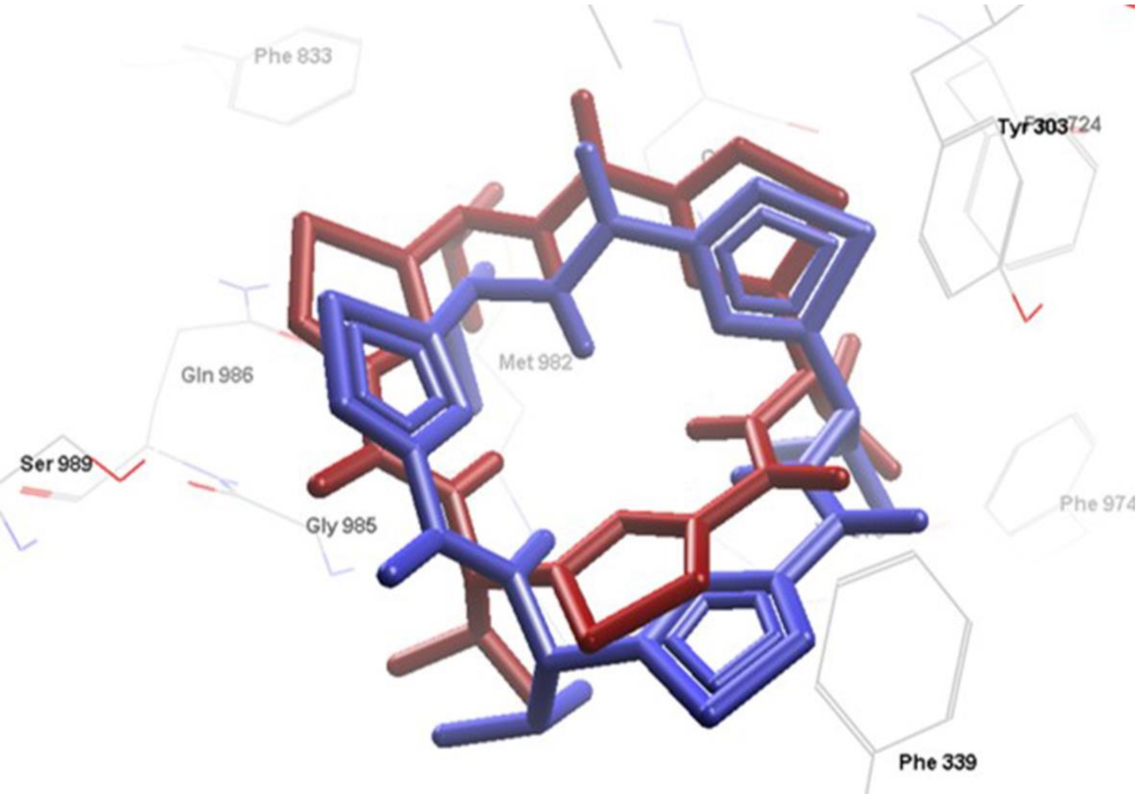

Figure S2. Structural validation of the docking calculations, using the crystal structure of mus musculus P-glycoprotein (PDB: 3G61).

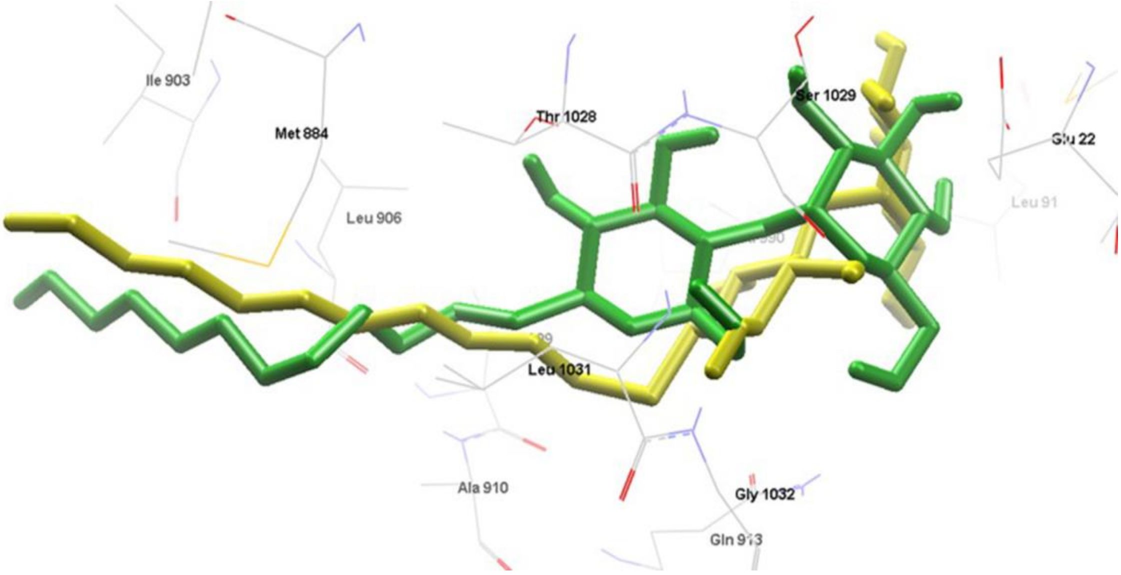

Figure S3. Structural validation of the docking calculations, using the crystal structure of Caenorhabditis elegans P-glycoprotein (PDB: 4F4C).

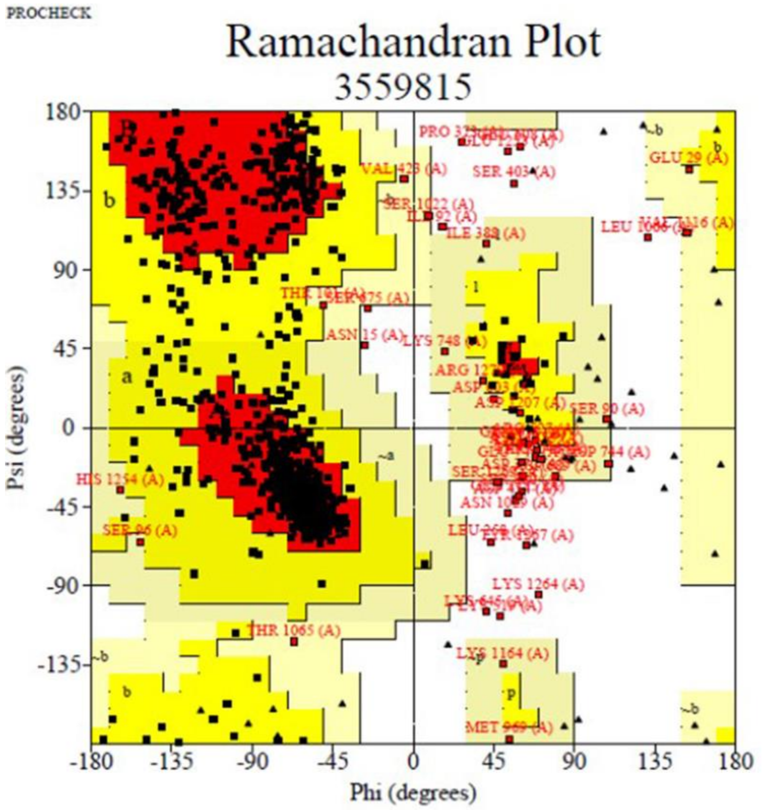

Plot statistics

Residues in most favoured regions [A,B,L]	970	84.3%
Residues in additional allowed regions [a,b,l,p]	138	12.0%
Residues in generously allowed regions [~a,~b,~l,~p]	26	2.3%
Residues in disallowed regions	17	1.5%
	----	------
Number of non-glycine and non-proline residues	1151	100.0%
Number of end-residues (excl. Gly and Pro)	2	
Number of glycine residues (shown as triangles)	98	
Number of proline residues	29	

Total number of residues	1280	

Figure S4. Ramachandran plot of mP-gp homology model.

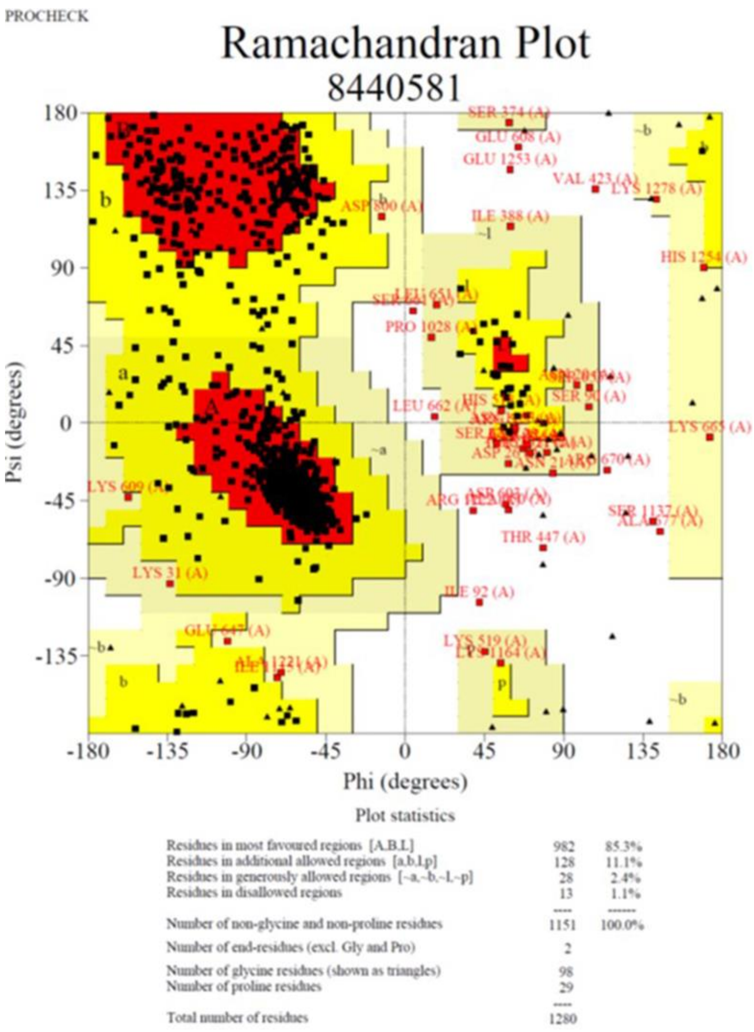

Figure S5. Ramachandran plot of cP-gp homology model.

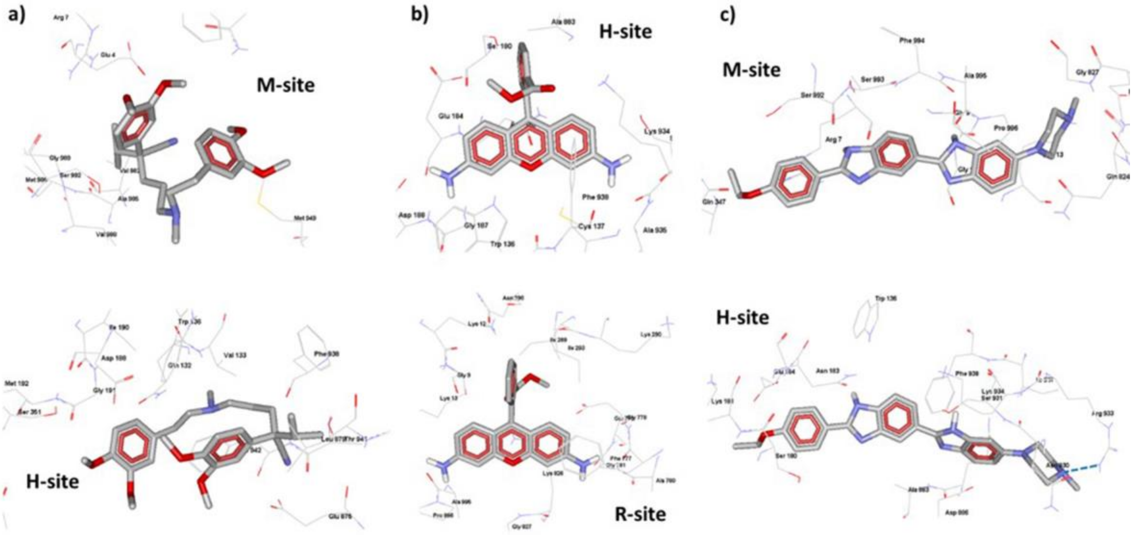

Figure S6. HB interactions of Verapamil, Rhodamine123 and Hoechst 33342 with mP-gp homology model.
